# Sex-specific trajectories of blood pressure and pulse pressure across body mass index categories: a descriptive study based on 13-year health checkup data

**DOI:** 10.1038/s41440-026-02607-7

**Published:** 2026-03-24

**Authors:** Shin Kawasoe, Takuro Kubozono, Yuichi Akasaki, Daisuke Tokutake, Shuya Shinchi, Satoko Ojima, Satoshi Yamaguchi, Satoshi Mukai, Hironori Miyahara, Koichi Tokushige, Masaaki Miyata, Mitsuru Ohishi

**Affiliations:** 1 Department of Cardiovascular Medicine and Hypertension, Graduate School of Medical and Dental Sciences, Kagoshima University, Kagoshima, Japan; 2 Kagoshima Kouseiren Hospital, Kagoshima, Japan; 3 School of Health Sciences, Faculty of Medicine, Kagoshima University, Kagoshima, Japan

**Keywords:** Aging, Blood pressure, BMI, Obesity, Sex differences

## Abstract

The combined effects of aging, sex, and body mass index (BMI) on blood pressure trajectories remain incompletely characterized in large-scale populations. The aim was to describe age-related blood pressure trajectories according to sex and BMI, as understanding these patterns is essential to improve risk stratification and develop preventive strategies for hypertension and cardiovascular diseases. This study included individuals who underwent annual physical examinations at Kagoshima Kouseiren Hospital in 2007–2019 (*n* = 213058). Systolic blood pressure (SBP), diastolic blood pressure (DBP), heart rate, and BMI measurements were retrospectively analyzed, and age-related changes were visualized using locally weighted scatterplot smoothing (LOWESS). Analyses were stratified by sex and BMI category (underweight, normal weight, class 1 obesity, and class 2 obesity or higher). Higher BMI levels were consistently accompanied by higher SBP, DBP, pulse pressure, and heart rate in both sexes. The LOWESS curves revealed that SBP increased steadily with age, particularly in individuals with obesity, whereas DBP increased until middle age before plateauing or declining. Consequently, the pulse pressure widened with age, especially in men with obesity. Heart rates remained stable but were consistently higher in women and obese participants. The age-related divergence between SBP and DBP was more pronounced in men and in individuals with a higher BMI. Ultimately, distinct age-related blood pressure and pulse pressure changes varied by sex and BMI category, and obesity was associated with accelerated SBP and pulse pressure trajectories, suggesting features consistent with earlier vascular aging. Adopting sex- and BMI-specific approaches may help inform blood pressure monitoring and prevention.

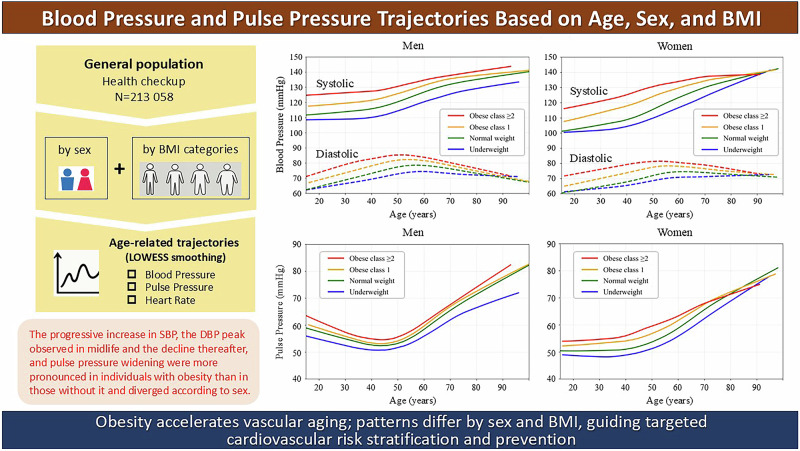

## Introduction

Hypertension is a leading modifiable risk factor for cardiovascular disease, and overcoming it remains a major public health challenge globally [1, 2]. The pathogenesis of hypertension is influenced by multiple factors, including aging, sex, and adiposity, which together are reflected in distinct hemodynamic trajectories across the lifespan [3, 4]. Among the key blood pressure components, systolic blood pressure (SBP) typically increases steadily with age, whereas diastolic blood pressure (DBP) tends to peak in midlife before plateauing or declining with age thereafter [4, 5]. This divergence leads to a progressive increase in the pulse pressure, which serves as a surrogate marker of arterial stiffness and is an independent predictor of cardiovascular events, especially in older adults [6].

Age-related SBP and DBP changes differ by sex owing to the influence of hormonal and vascular factors, and they may be further modified by adiposity, which is commonly measured using the body mass index (BMI), a well-established correlate of elevated blood pressure through mechanisms that include increased sympathetic activity, sodium retention, and endothelial dysfunction [7, 8]. However, few large population-based studies have simultaneously visualized age-dependent SBP and DBP trajectories stratified by both sex and BMI categories, particularly in East Asian populations in which BMI distributions and cardiovascular risk profiles differ from those in Western populations [2].

In addition to SBP and DBP, the resting heart rate (HR), which reflects autonomic regulation and metabolic demand, may also vary with age and the degree of obesity [9]. Concurrent assessment of SBP, DBP, pulse pressure, and HR can provide a more nuanced understanding of vascular aging and differences in the hemodynamic burden across demographic strata.

The aim of this study was to elucidate the age-related trajectories of SBP, DBP, pulse pressure, and HR according to sex and BMI categories using health checkup data collected over a 13-year period in a Japanese cohort. Locally weighted scatterplot smoothing (LOWESS) was applied to generate detailed visualizations of hemodynamic aging patterns and may help inform risk stratification and early prevention in clinical and public health settings.

Point of view

- **Clinical relevance**
  Sex- and BMI-specific blood pressure trajectories may improve individualized interpretation of SBP/DBP components and support earlier risk communication in health checkup practice.
- **Future direction**
  Longitudinal modeling that accounts for within-person correlation and incorporates lifestyle and menopausal status is warranted to quantify determinants of trajectory inflection points.
- **Consideration for the Asian population**
  In East Asians, cardiometabolic risk can rise at relatively modest BMI levels; therefore, sex- and adiposity-stratified monitoring may be particularly important in Asian screening systems.

## Methods

### Study design and population

This retrospective descriptive study was conducted using data collected from individuals who had undergone annual health checkups at Kagoshima Kouseiren Hospital between April 2007 and March 2019. All examinees participated in a standardized health screening program that included anthropometric measurements, blood pressure assessments, and blood sampling. Individuals with missing data for key variables such as age, sex, SBP, DBP, BMI, or HR were excluded. To describe age-related trends, all available checkup records from the 13-year dataset were utilized, allowing for multiple observations per person.

### Measurements

Trained medical staff performed health checkups in accordance with standard procedures. Briefly, blood pressure was measured using an automated oscillometric device in a seated position after the participant had remained at rest for at least five minutes. SBP and DBP were recorded in mmHg, and the pulse pressure was calculated as the SBP minus the DBP. The resting HR (beats per minute) was not directly recorded during the participants’ health checkups; therefore, the HR obtained from a resting 12-lead electrocardiogram (ECG) was used as a substitute. The ECG was performed in a supine position. Although transient irregular rhythms or motion artifacts may have caused minimal variability, these effects were expected to be negligible at the population level.

Height and weight were measured using calibrated equipment, and each participant’s BMI was calculated as the weight in kilograms divided by the height in meters squared (kg/m²). Based on the Japan Society for the Study of Obesity classification guidelines, participants were categorized into the following four BMI groups: underweight (<18.5 kg/m²), normal weight (18.5–24.9 kg/m²), obese class 1 (25.0–29.9 kg/m²), and obese class 2 or higher (≥30.0 kg/m²).

A self-administered questionnaire was used to obtain information on drugs used for hypertension, dyslipidemia, and diabetes. Blood samples were collected after overnight fasting. Serum lipid, glucose, uric acid, and creatinine levels were measured using standard laboratory procedures. Hypertension was defined as SBP of 140 mmHg or higher, DBP of 90 mmHg or higher, or those taking antihypertensive medication, according to guidelines published in 2019 by the Japanese Society of Hypertension.

### Statistical analysis

We summarized baseline characteristics of participants by age group and sex as Table 1. Characteristics included anthropometric indices, blood pressure values, metabolic and renal function-related values obtained from blood tests, HR from a resting 12-lead electrocardiogram, and the proportion of those receiving medication for hypertension, dyslipidemia, and diabetes. Age was categorized in 5-year increments, and only data from the earliest visit were used for those who had multiple visits during that time period. This method has the limitation that the age of data acquisition is different for each individual within an age category and that it tends to be biased toward data from earlier age periods within a category. On the other hand, it has the advantage of preventing weighted effects due to multiple contributions by the same person, and is considered suitable for presenting baseline tabulations. In contrast, for the visualization of age-related hemodynamic trends using LOWESS, all available measurements were utilized so that each participant could contribute to multiple age categories if applicable. This approach was adopted to ensure continuity of data across the entire age range and improve the stability of the smoothed curves, while maintaining the analysis at the population level rather than the individual level.

**Table 1:** Baseline characteristics of the study population by sex and age group

Male														
Age, years	≤24	25–29	30–34	35–39	40–44	45–49	50–54	55–59	60–64	65–69	70–74	75–79	80–84	≥85
Number of participants	5829	7566	9417	11837	13883	15127	17086	20030	23250	22846	18735	12109	6645	2328
BMI, kg/m^2^	22.5 (4.1)	23.3 (4.1)	23.8 (4.1)	24.1 (4.0)	24.3 (3.8)	24.3 (3.6)	24.1 (3.4)	23.9 (3.2)	23.7 (3.0)	23.7 (2.9)	23.5 (2.9)	23.2 (2.9)	22.9 (3.0)	22.4 (3.0)
underweight (<18.5 kg/m²), %	11.7	6.8	4.8	3.7	2.9	2.5	2.7	2.9	2.9	3.0	3.4	4.1	5.7	8.4
normal weight (18.5–24.9 kg/m²), %	66.1	64.9	62.9	61.1	58.8	58.5	59.5	62.1	65.6	66.5	66.8	68.6	70.5	72.6
obese class 1 (25.0–29.9 kg/m²), %	15.7	20.8	23.8	26.7	29.9	31.2	32.2	30.7	28.3	27.8	27.8	25.4	22.2	18.0
obese class 2 or higher (>=30.0 kg/m²), %	6.6	7.5	8.4	8.4	8.4	7.8	5.6	4.3	3.2	2.7	2.0	1.8	1.6	1.0
Waist circumference, cm	77.9 (10.4)	81.5 (10.8)	83.5 (10.5)	84.5 (10.5)	85.5 (10.1)	86.0 (9.5)	85.9 (9.0)	85.6 (8.5)	85.1 (8.3)	85.0 (8.2)	84.8 (8.3)	84.6 (8.4)	84.2 (8.4)	82.8 (8.6)
SBP, mmHg	114.4 (11.6)	115.4 (11.7)	116.2 (12.8)	117.3 (14.0)	119.2 (14.9)	121.6 (15.8)	124.7 (16.7)	127.9 (17.4)	130.7 (17.5)	133.5 (17.4)	135 (17.3)	135.8 (17.3)	136.8 (17.4)	137.3 (18.0)
DBP, mmHg	66.4 (9.6)	69.1 (9.8)	71.6 (10.7)	74.1 (11.5)	76.6 (11.8))	79.0 (12.0))	80.3 (11.5)	80.6 (11.2)	79.7 (10.7)	78.7 (10.5)	77.1 (10.3)	75.2 (10.4)	73.6 (10.7)	72.3 (10.9)
Triglyceride, mg/dL	69 (50, 99)	83 (59, 126)	95 (66, 145)	107 (72, 165)	114 (77, 177)	117 (80, 181)	114 (79, 175)	109 (76, 163)	102 (72, 151)	97 (70, 140)	92 (67, 129)	86 (64, 120)	83 (63, 115)	81 (61, 108)
HDL-C, mg/dL	56.6 (11.9)	55.8 (12.6)	55.0 (13.0)	54.9 (13.8)	55.1 (14.3)	55.6 (14.4)	56.3 (14.7)	57.2 (15.0)	57.6 (14.9)	57.6 (14.8)	57.1 (14.4)	56.8 (14.1)	56.3 (14.0)	55.8 (14.1)
LDL-C, mg/dL	100.3 (27.2)	109.1 (29.2)	115.3 (31.0)	119.8 (32.3)	122.8 (33.0)	124.2 (33.3)	123.0 (32.5)	121.4 (31.9)	118.6 (30.7)	117.0 (29.8)	115.1 (28.5)	112.9 (27.4)	111.1 (27.2)	109.1 (26.6)
FBS, mg/dL	91 (86, 96)	91 (86, 96)	92 (87, 98)	94 (89, 100)	95 (90, 102)	97 (91, 105)	99 (92, 108)	100 (93, 111)	100 (93, 111)	100 (92, 111)	99 (92, 110)	99 (92, 109)	98 (91, 107)	97 (91, 106)
HbA1c (NGSP), %	4.9 (0.6)	4.9 (0.4)	5.0 (0.5)	5.1 (0.6)	5.2 (0.7)	5.3 (0.9)	5.4 (0.9)	5.5 (0.9)	5.5 (0.9)	5.5 (0.8)	5.5 (0.8)	5.5 (0.7)	5.5 (0.7)	5.4 (0.6)
UA, mg/dL	6.0 (1.2)	6.1 (1.2)	6.2 (1.3)	6.2 (1.3)	6.2 (1.3)	6.1 (1.3)	6.1 (1.4)	6.0 (1.4)	6.0 (1.3)	6.0 (1.3)	5.9 (1.3)	5.8 (1.3)	5.8 (1.4)	5.8 (1.4)
Creatinine, mg/dL	0.83 (0.11)	0.84 (0.19)	0.83 (0.16)	0.83 (0.18)	0.83 (0.26)	0.84 (0.31)	0.84 (0.35)	0.84 (0.34)	0.85 (0.32)	0.86 (0.32)	0.87 (0.24)	0.89 (0.28)	0.93 (0.30)	0.99 (0.32)
eGFR	100.2 (14.7)	94.2 (13.5)	90.1 (13.1)	86.7 (13.1)	83.9 (13.5)	81.4 (14.0)	79.2 (14.1)	77.3 (14.4)	74.5 (14.6)	72.2 (14.4)	69.6 (14.3)	67.0 (14.6)	63.3 (14.6)	59.3 (15.0)
Heart rate, /min	64.8 (10.7)	64.4 (10.1)	64.1 (9.9)	63.8 (9.8)	64.0 (10.1)	64.3 (10.3)	64.2 (10.5)	63.8 (10.5)	63.2 (10.4)	63.0 (10.5)	62.8 (10.7)	63.3 (11.1)	64.4 (11.5)	66.3 (11.9)
Medication for hypertension, %	0.1	0.2	0.7	1.9	4.9	10.1	16.8	24.3	32.4	39.9	45.5	50.6	52.9	55.4
Medication for dyslipidemia, %	0.1	0.2	0.4	0.8	2.1	3.8	6.2	8.2	10.7	12.5	13.7	14.2	14.0	13.7
Medication for diabetes, %	0.1	0.3	0.3	0.8	1.6	3.1	5.1	6.8	8.4	9.8	10.8	10.6	9.9	9.2
Female														
Age, years	≤24	25–29	30–34	35–39	40–44	45–49	50–54	55–59	60–64	65–69	70–74	75–79	80–84	≥85
Number of participants	6284	6375	7397	9484	11836	13293	16419	21070	25003	25637	22463	14766	7586	2475
BMI, kg/m^2^	20.9 (3.5)	21.1 (3.8)	21.5 (4.0)	21.9 (4.1)	22.3 (4.0)	22.7 (3.9)	22.9 (3.7)	22.9 (3.5)	23.0 (3.5)	23.2 (3.4)	23.2 (3.4)	23.1 (3.4)	22.9 (3.4)	22.5 (3.4)
underweight (<18.5 kg/m²), %	21.8	22.2	19.0	15.6	11.9	9.3	8.4	8.1	6.9	6.7	6.7	7.0	7.7	11.0
normal weight (18.5–24.9 kg/m²), %	68.6	64.1	65.6	66.5	67.5	66.6	67.3	67.8	67.9	67.4	66.7	66.3	66.2	65.7
obese class 1 (25.0–29.9 kg/m²), %	7.1	10.2	10.9	12.4	15.2	18.2	19.3	19.8	21.3	22.3	23.1	23.6	23.1	20.5
obese class 2 or higher (>=30.0 kg/m²), %	2.5	3.5	4.5	5.5	5.4	5.9	5.0	4.3	4.0	3.7	3.5	3.1	3.0	2.8
Waist circumference, cm	73.1 (8.6)	75.3 (9.2)	76.6 (9.6)	77.9 (10.2)	79.1 (10.1)	80.3 (10.0)	81.3 (9.8)	82.1 (9.5)	82.7 (9.4)	83.4 (9.4)	83.7 (9.6)	83.9 (9.3)	83.4 (9.5)	82.1 (10.0)
SBP, mmHg	104.8 (11.1)	104.7 (11.3)	106.0 (12.2)	107.9 (13.6)	110.9 (15.2)	115.9 (17.0)	120.4 (17.9)	123.7 (17.7)	127.1 (17.7)	130.1 (17.5)	133.2 (17.4)	135.1 (17.0)	136.4 (17.0)	138.4 (17.9)
DBP, mmHg	63.4 (8.9)	64.5 (9.1)	65.8 (9.7)	67.7 (10.5)	69.8 (11.3)	72.6 (11.6)	75.1 (11.5)	75.8 (11.0)	76.0 (10.8)	75.6 (10.4)	75.0 (10.4)	74.1 (10.2)	73.4 (10.5)	73.4 (10.9)
Triglyceride, mg/dL	53 (41, 71)	55 (42, 74)	57 (44, 78)	60 (46, 84)	64 (48, 89)	69 (52, 97)	77 (57, 108)	82 (61, 114)	86 (64, 119)	89 (66, 121)	88 (67, 119)	89 (67, 119)	89 (68, 117)	88 (67, 117)
HDL-C, mg/dL	65.7 (12.9)	66.7 (13.3)	66.5 (13.8)	66.4 (14.0)	66.3 (14.3)	66.7 (14.9)	67.3 (15.4)	66.4 (15.4)	64.7 (14.9)	63.3 (14.4)	62.4 (14.1)	61.6 (13.9)	60.9 (14.0)	60.5 (14.1)
LDL-C, mg/dL	96.6 (24.6)	98.7 (25.7)	101.3 (27.2)	105.3 (27.8)	110.1 (28.6)	118.0 (30.1)	128.8 (31.7)	133.6 (31.3)	132.3 (30.5)	129.8 (29.8)	125.4 (28.7)	122.5 (27.7)	119.9 (27.2)	117.0 (27.4)
FBS, mg/dL	87 (83, 91)	86 (82, 91)	87 (83, 92)	89 (84, 94)	90 (85, 95)	91 (86, 97)	92 (87, 99)	93 (88, 100)	94 (88, 101)	94 (88, 102)	94 (89, 102)	94 (89, 102)	94 (89, 102)	94 (89, 102)
HbA1c (NGSP), %	4.9 (0.3)	4.9 (0.3)	4.9 (0.3)	5.0 (0.4)	5.1 (0.5)	5.2 (0.5)	5.3 (0.6)	5.4 (0.6)	5.4 (0.7)	5.5 (0.7)	5.5 (0.6)	5.5 (0.6)	5.4 (0.6)	5.4 (0.6)
UA, mg/dL	4.4 (0.9)	4.3 (1.0)	4.3 (1.0)	4.2 (1.0)	4.2 (1.0)	4.2 (1.0)	4.4 (1.0)	4.6 (1.0)	4.6 (1.1)	4.6 (1.1)	4.7 (1.1)	4.7 (1.2)	4.8 (1.2)	5.0 (1.3)
Creatinine, mg/dL	0.61 (0.09)	0.60 (0.09)	0.60 (0.09)	0.61 (0.09)	0.61 (0.14)	0.61 (0.15)	0.62 (0.18)	0.62 (0.16)	0.63 (0.14)	0.64 (0.19)	0.65 (0.16)	0.67 (0.17)	0.71 (0.21)	0.75 (0.23)
eGFR	105.4 (17.2)	99.7 (16.5)	95.1 (16.4)	90.0 (14.6)	86.3 (14.0)	83.7 (14.0)	81.1 (14.1)	78.6 (13.9)	75.9 (13.6)	73.5 (13.7)	70.6 (13.8)	67.3 (14.0)	63.1 (14.0)	59.0 (14.4)
Heart rate, /min	68.7 (10.7)	67.5 (10.2)	66.8 (9.7)	66.6 (9.6)	66.5 (9.8)	66.9 (10.0)	66.3 (10.0)	65.8 (9.8)	66.0 (9.9)	66.1 (10.0)	66.7 (10.3)	67.5 (10.6)	68.8 (10.8)	71.1 (12.0)
Medication for hypertension, %	0.1	0.0	0.3	0.9	2.3	5.5	11.0	17.9	25.2	33.7	43.0	51.7	58.3	61.3
Medication for dyslipidemia, %	0.0	0.0	0.2	0.4	0.7	1.7	4.4	9.3	15.7	20.3	24.7	26.1	25.1	23.2
Medication for diabetes, %	0.1	0.1	0.3	0.4	0.6	1.0	2.0	2.9	4.2	5.5	6.3	6.4	6.9	5.9

Baseline characteristics, including body mass index (BMI), waist circumference, systolic blood pressure (SBP), diastolic blood pressure (DBP), resting heart rate, and laboratory measurements, were summarized separately for men and women in each 5-year age group. The proportion of individuals who had received treatment for hypertension, dyslipidemia, and diabetes is also reported. Only the earliest available records were used when multiple measurements were available for the same individual in the same age group. Triglyceride and fasting blood glucose were shown as medians (first and third quartiles); other numerical data were shown as means (standard deviations). The percentage of medically treated patients for the disease was shown as a percentage

*TG* triglyceride, *HDL-C* high-density lipoprotein cholesterol, *LDL-C* low-density lipoprotein cholesterol, *FBS* fasting blood glucose, *UA* uric acid, *eGFR* estimated glomerular filtration rate

**Fig. 1:**
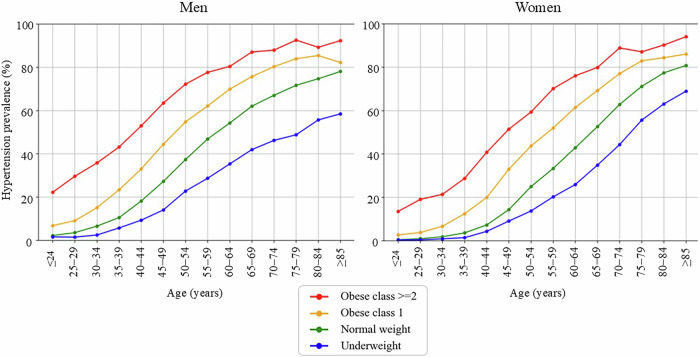
Prevalence of hypertension in each age group by gender and BMI category. Hypertension was defined as systolic blood pressure (SBP) ≥ 140 mmHg, diastolic blood pressure (DBP) ≥ 90 mmHg, or under treatment with antihypertensive medication. Data are shown for 5-year age intervals. Those who had multiple visits during the period were counted as having hypertension if they met the definition at least once. Body mass index (BMI) categories were defined as follows: underweight, <18.5 kg/m²; normal weight, 18.5–24.9 kg/m²; obese class 1, 25.0–29.9 kg/m²; and obese class 2 or higher, ≥30.0 kg/m²

We then tabulated the prevalence of hypertension in each age group by gender and BMI category and presented a line graph as Fig. 1. At this point, those who had multiple visits during the period were counted as having hypertension if they met the definition at least once. This approach was adopted to ensure that individuals with borderline blood pressure values were not overlooked, which is consistent with a preventive medicine perspective.

To visualize age-related trends in hemodynamic parameters, LOWESS was applied to the SBP, DBP, pulse pressure, and HR data. LOWESS is a non-parametric regression technique originally introduced by Cleveland in 1979 [10, 11] that constructs a smooth curve by fitting simple regression models in the neighborhood of each target data point. Because some individuals contributed multiple observations across different age categories, repeated measurements from the same participant were treated as independent data points. The LOWESS method does not explicitly account for within-individual correlations; therefore, the generated curves should be interpreted as population-level smoothed trends rather than subject-level longitudinal changes. Accordingly, the analysis was intended to illustrate population-level patterns rather than to estimate independent or causal effects. Unlike global regression models, LOWESS does not assume that a predefined relationship exists between variables, and the technique is well-suited for identifying complex and potentially non-linear patterns in observational data.

When conducting LOWESS, each localized fit was performed using a weighted least squares approach, with the weight of each data point determined by its distance from the focal point and assigned according to the tricube weight function:

$${{{{\rm{w}}}}({{{\rm{x}}}})=({1-|{{{\rm{d}}}}|}^{3})}^{3}\,{{{\rm{for}}}}|{{{\rm{d}}}}| < 1=0\,{{{\rm{otherwise}}}}$$

where d represents the normalized distance between the predictor value of a neighboring point and that of the target point.

**Fig. 2:**
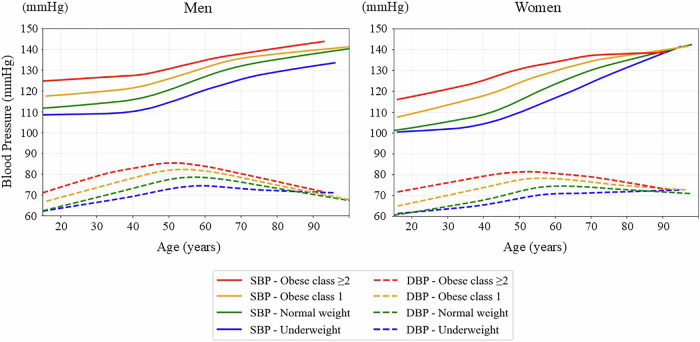
LOWESS curves of systolic and diastolic blood pressure by age, sex, and BMI category. The panels show population-level, cross-sectional visualizations of the systolic blood pressure (SBP) and diastolic blood pressure (DBP) trajectories using locally weighted scatterplot smoothing (LOWESS) for men (left) and women (right). The curves are stratified by body mass index (BMI) category as follows: underweight, <18.5 kg/m²; normal weight, 18.5–24.9 kg/m²; obese class 1, 25.0–29.9 kg/m²; and obese class 2 or higher, ≥30.0 kg/m². Solid lines represent SBP, whereas dashed lines represent DBP. The curves represent population-level smoothed trends rather than individual longitudinal trajectories

**Fig. 3:**
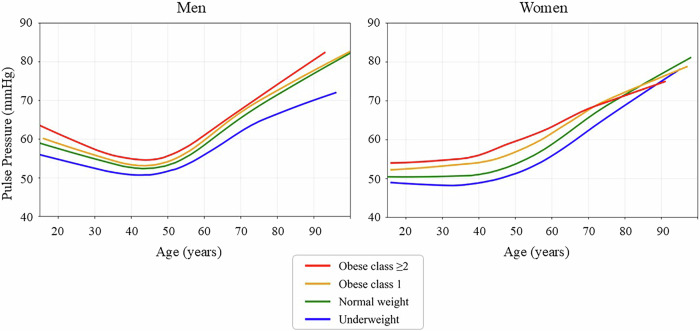
LOWESS curves of pulse pressure by age, sex, and BMI category. The panels show population-level, cross-sectional visualizations of the age-related pulse pressure trajectories using locally weighted scatterplot smoothing (LOWESS) by sex (men: left; women: right) and BMI category. The curves are stratified by body mass index (BMI) category as follows: underweight, <18.5 kg/m²; normal weight, 18.5–24.9 kg/m²; obese class 1, 25.0–29.9 kg/m²; and obese class 2 or higher, ≥30.0 kg/m². The curves represent population-level smoothed trends rather than individual longitudinal trajectories

**Fig. 4:**
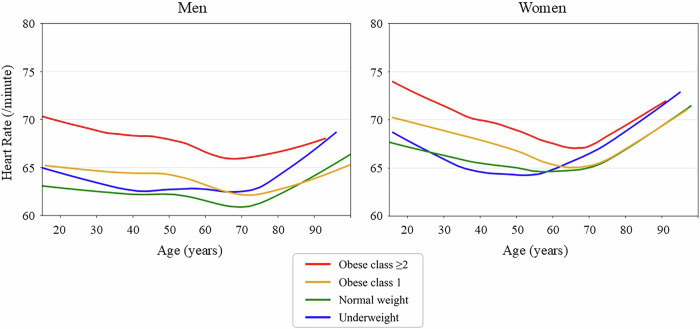
LOWESS curves of heart rate by age, sex, and BMI category. The panels show population-level, cross-sectional visualizations of the age-related resting heart rate trajectories using locally weighted scatterplot smoothing (LOWESS) by sex (men: left; women: right) and BMI category. The curves are stratified by body mass index (BMI) category as follows: underweight, <18.5 kg/m²; normal weight, 18.5–24.9 kg/m²; obese class 1, 25.0–29.9 kg/m²; and obese class 2 or higher, ≥30.0 kg/m². The curves represent population-level smoothed trends rather than individual longitudinal trajectories

The tricube weight function ensures that points closer to the target are weighted more heavily, whereas more distant points contribute less or are excluded. For all LOWESS curves, a local linear model (first-degree polynomial) was used for local fitting, which is the default and most commonly used approach for epidemiological applications. This method effectively captures gradual changes over time without overfitting. Smoothing was performed separately by sex and BMI category. The smoothing span parameter (frac) was set to 0.2 to balance the curve’s smoothness with the sensitivity to local variations, based on empirical testing. The curves of SBP and DBP, pulse pressure, and HR by sex and by BMI category are shown in Figs. 2, 3, and 4, respectively.

Previous studies have shown that SBP consistently increases with age, whereas DBP increases in youth and middle age and declines thereafter; to estimate the age at which DBP goes from increasing to declining with age, a quadratic polynomial regression for each gender and BMI category (DBP ~Age + Age ²) was applied; peak age for DBP was calculated as the vertex of the fitted parabola, and confidence intervals were estimated using the delta method. The results of this method, which quantitatively assesses the DBP tipping point statistically without relying on visual assessment, are presented in Table 2.

**Table 2:** Age at which DBP begins to decline by sex and BMI category

Sex	BMI_category	Age of DBP peak (95% CI)	Coefficient for age	Coefficient for age squared	R-squared
Male	Underweight	63.6 (62.4, 64.8)	0.7913	–0.00622	0.095
	Normal weight	58.6 (58.4, 58.7)	1.2574	–0.01073	0.099
	Obese class 1	54.9 (54.8, 55.1)	1.3731	–0.01250	0.078
	Obese class ≥2	51.0 (50.6, 51.5)	1.2671	–0.01242	0.064
Female	Underweight	86.2 (81.7, 90.8)	0.4541	–0.00263	0.104
	Normal weight	67.1 (66.7, 67.4)	0.8815	–0.00657	0.093
	Obese class 1	58.9 (58.6, 59.2)	0.9850	–0.00836	0.039
	Obese class ≥2	53.3 (52.6, 54.0)	0.8223	–0.00772	0.030

This table shows the estimated age at which diastolic blood pressure (DBP) reaches its maximum based on quadratic regression modeling. Analyses were stratified by sex and body mass index (BMI) category (underweight, <18.5 kg/m²; normal weight, 18.5–24.9 kg/m²; obese class 1, 25.0–29.9 kg/m²; and obese class 2 or higher, ≥30.0 kg/m²). The estimates represent the vertex of the fitted quadratic curve for each subgroup, with corresponding 95% confidence intervals derived using the delta method

**Fig. 5:**
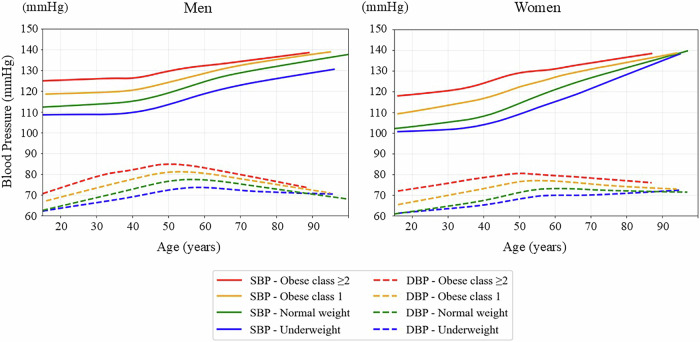
LOWESS curves of systolic and diastolic blood pressure by age, sex, and BMI category (excluding individuals being treated with antihypertensive medications). The panels show population-level, cross-sectional visualizations of the systolic blood pressure (SBP) and diastolic blood pressure (DBP) trajectories after excluding individuals who had received pharmacotherapies, regardless of their antihypertensive treatment status. Solid lines represent SBP, and dashed lines represent DBP across different body mass index (BMI) categories. The same smoothing approach and stratification used in Fig. 1 were applied. The curves represent population-level smoothed trends rather than individual longitudinal trajectories

To assess the potential modifying effects of antihypertensive therapy on the trajectories being investigated, the same LOWESS analysis was repeated after excluding participants who were being treated with antihypertensive medications at the time of the health checkup. The resulting curves (Fig. 5) were compared with those from the overall population (Fig. 2) to evaluate potential differences in age-related blood pressure patterns due to treatment.

Data management, statistical analysis, and visualization were performed using Python version 3.11. More specifically, the pandas library was used for data processing, statsmodels was employed for LOWESS fitting, and matplotlib was utilized for plotting.

## Results

### Baseline characteristics

A total of 213,058 unique individuals were confirmed to be eligible for inclusion based on their health checkup records, 108,217 (50.8%) of whom were men and 104,841 (49.2%) of whom were women. Table 1 presents the baseline characteristics of the study population, stratified by sex and age. The overall mean age was 47.8 ± 13.6 years in men and 49.2 ± 13.4 years in women. According to their BMI, 23.5% of men and 17.8% of women were classified as having class 1 obesity, whereas the proportions of men and women classified as having class 2 obesity or higher were 4.2% and 3.5%, respectively. Because the dataset consisted of repeated health checkup records collected at different time points, the results presented below describe age-related patterns at the population level rather than longitudinal changes within the same individuals.

### Hypertension prevalence

The prevalence of hypertension in each age group by gender and BMI category is shown in Fig. 1. Hypertension prevalence increased steadily with age across all BMI categories and in both sexes. Obese individuals consistently exhibited higher prevalence compared to normal weight and underweight counterparts, with differences becoming more pronounced in midlife and older adulthood. In men, the prevalence in the obese class ≥2 group surpassed 80% by age 60–64 and reached over 90% by age 75–79. In women, the obese class ≥2 group also showed a sharp rise in prevalence, reaching nearly 90% in the oldest age group. Underweight individuals had the lowest hypertension prevalence across all age groups. The disparity in hypertension prevalence by BMI widened with age, suggesting an amplifying effect of obesity on age-related blood pressure elevation.

### Age-related trends in SBP and DBP

Figure 2 shows the age-related, sex-stratified SBP and DBP trajectories across BMI categories using the LOWESS method, illustrating population-level, cross-sectional patterns across age groups rather than individual longitudinal trajectories. In both men and women, the SBP increased consistently and gradually with age. Higher BMI categories were associated with SBP elevation, beginning at a young age and persisting into middle age and older adulthood. The slope of the increasing SBP curve also became steeper at a younger age in the groups with obese class 1 and class 2 or higher, indicating that those participants experienced an earlier vascular burden onset. Within the same BMI category, men exhibited a higher SBP than women did in the 20–40-year age group. However, the slope of the increasing SBP curve became steeper in women after midlife, particularly among those in the higher BMI categories, resulting in SBP levels in older women that were comparable to or even exceeded those in men.

In contrast, DBP followed a nonlinear trajectory; more specifically, it increased until approximately the fifth decade of life before gradually declining. This late-life decline was more pronounced in men than in women and was steeper in the higher BMI categories than in the lower ones in both sexes. Table 2 shows the estimated age at which DBP begins to decline by gender and BMI category. The age of DBP peaked varied by sex and BMI category. In men, the DBP peak occurred earlier than in women, and individuals with higher BMI showed younger peak ages. For example, among men with normal weight, the DBP peaked at 58.6 years (95% CI: 58.4–58.7), while in obese class ≥2 women, the peak occurred as early as 53.3 years (95% CI: 52.6–54.0). Conversely, underweight women exhibited a substantially delayed DBP peak at 86.2 years (95% CI: 81.7–90.8). These results support the notion that obesity accelerates vascular aging, leading to an earlier transition from diastolic rise to decline.

### Age-related trends in pulse pressure and HR

Figure 3 presents the age-related trajectories of pulse pressure stratified by BMI category and sex, while Fig. 4 shows the corresponding trajectories of resting HR. In men, the pulse pressure initially declined during early adulthood, reaching a plateau at 40–49 years before increasing continuously with advancing age. In contrast, in women, the pulse pressure remained relatively stable until their 30 s and began to rise thereafter. The slope of the curve for this age-related increase was especially steep in individuals with class 1 obesity and class 2 obesity or higher, and those participants began to exhibit elevated pulse pressure levels in midlife. The age at which the pulse pressure began to rise did not differ between the BMI categories for men, but tended to occur earlier for women in the higher BMI categories. Differences in the pulse pressure between the BMI categories were greater in women than in men.

The resting HR plateaued or gradually decreased from early to mid-adulthood but increased markedly after age 70 in both sexes. At all ages, the HR elevation was more pronounced in individuals with a greater BMI, with the highest values observed in those with an obesity class 2 or higher. This BMI-related difference in HR was more pronounced in women than in men. Across all age groups and BMI strata, women consistently exhibited a higher resting HR than men did, and the combination of female sex and obesity was associated with the highest HR elevation.

### Additional analysis after excluding individuals receiving pharmacotherapy

Figure 5 illustrates the SBP and DBP age-related trajectories in individuals who did not receive antihypertensive medications. Compared to Fig. 2, which shows pooled data for both treated and untreated individuals, the overall patterns after excluding those participants remained highly consistent, with the SBP progressively increasing with advancing age and the DBP following a characteristic nonlinear trajectory, peaking in midlife and declining thereafter.

The influence of the BMI remained evident across all age groups and both sexes, regardless of the participants’ treatment status. Individuals with obesity consistently exhibited higher SBP and DBP values compared to those in their normal weight counterparts, and the divergence between SBP and DBP (i.e., pulse pressure) became more pronounced with age, especially in the higher BMI categories. While SBP levels were slightly lower in older untreated individuals, particularly among those with obese class 2 or higher, the age-related widening of the pulse pressure remained clearly observable.

The previously observed sex-based differences were also retained in the untreated population. In younger age groups, women had exhibited a lower SBP than men did; however, this difference diminished or was reversed in older age, particularly among individuals with obesity, mirroring the patterns observed in the full cohort. These findings underscore the robust contributions of age, sex, and adiposity to blood pressure trajectories, even in the absence of antihypertensive therapy.

## Discussion

This large-scale study based on 13 years of health check-up data from a Japanese cohort investigated age-related changes in SBP, DBP, pulse pressure, and resting HR trajectories, with stratification by sex and BMI categories. Using the LOWESS technique, continuous hemodynamic changes across the adult lifespans of both men and women were plotted.

The findings provide several insights. First, the SBP increased progressively with age in both sexes and all BMI groups, with notably steeper slopes in individuals with obesity. This trend is consistent with that of previous studies demonstrating age-associated arterial stiffening and increased peripheral resistance [5, 12]. Second, the DBP followed a nonlinear, biphasic curve, peaking in midlife and declining thereafter, and this trajectory was more pronounced in men than in women and in those with a higher BMI, thereby reflecting sex-specific hormonal influences and age-related reductions in vascular tone [13–15]. The earlier DBP peak observed in the participants in the higher BMI categories was indicative of accelerated vascular aging in these populations [16]. Although arterial stiffness may contribute to a decline in DBP in older adults, our findings showed that DBP remained relatively higher in obese individuals even in later life. This may be partly explained by non-vascular factors such as increased circulating blood volume and cardiac output in obesity. Obese individuals typically have higher salt intake and expanded extracellular fluid volume, which may sustain elevated DBP despite advancing vascular aging [17, 18]. Third, the divergence between SBP and DBP with age resulted in a widening pulse pressure, particularly in individuals with obesity. In men, the pulse pressure slightly declined during early adulthood, reaching a minimum at 40–49 years before rising steeply with advancing age. In contrast, in women, the pulse pressure remained relatively stable until their 30 s and began to increase thereafter. The earlier onset of pulse pressure elevation in women compared with men is likely due to hormonal changes that occur around the time of menopause, especially estrogen decline, which contributes to arterial stiffening and increases the SBP [19]. In contrast, the steeper pulse pressure increase observed in men post-inflection may reflect a faster progression of arterial stiffness owing to accumulated cardiovascular risk factors, such as smoking, dyslipidemia, and physical inactivity. In addition, in men with obesity, increased cardiac output and peripheral resistance are likely to exacerbate these trends. The widening gap between the SBP and DBP that occurred with age, especially in individuals with obesity, underscores the role of adiposity in accelerating vascular aging, potentially through mechanisms such as endothelial dysfunction and sympathetic overactivity [20–22].

These observations are consistent with the findings from both the Framingham Heart Study and the Ohasama Study, which described similar SBP-DBP divergence patterns in Western and East Asian populations, respectively [5, 23]. While these general trends have been observed previously [24], the present study adds important new evidence by integrating sex- and BMI-stratified analyses within a large Japanese cohort and by using a non-parametric smoothing technique to capture continuous, nonlinear hemodynamic trajectories across adulthood. This approach enabled identification of population-specific inflection points—such as the earlier DBP peak and pulse pressure widening in obese individuals and in women—that have not been systematically described in Asian populations, thereby extending the understanding of vascular aging dynamics beyond prior studies. The difference in data handling between Table 1 and the LOWESS analyses reflects their distinct analytical purposes: Table 1 describes the baseline characteristics of the cohort using the first available record per participant to avoid weighting bias, whereas the LOWESS analysis includes all available measurements to depict continuous, population-level age-related patterns across the adult lifespan.

The resting HR remained relatively stable throughout adulthood but increased after 70 years of age in both sexes. This late-life elevation may have reflected an increasing prevalence of atrial fibrillation, diminished parasympathetic tone, or heightened cardiovascular stress [25, 26]. Across all ages, the HR was consistently higher in those with obesity, particularly in women. The combination of female sex and obesity was associated with the greatest HR elevation, possibly reflecting the synergistic effects of adiposity and sex-specific autonomic regulation [27]. These findings highlight the importance of considering HR as an additional marker of cardiovascular stress and metabolic load.

The supplementary analysis, which excluded the individuals who had received antihypertensive therapy, confirmed the robustness of these patterns. The age-related SBP and DBP trajectories were largely preserved, suggesting that antihypertensive treatment may lower absolute blood pressure values without fundamentally altering the underlying hemodynamic aging process [28]. The persistent BMI gradient across all metrics reinforces the strong and independent influence of adiposity on vascular health described in other studies [16,29,30]. The similarity in trends between the treated and untreated groups emphasizes the physiological basis of vascular aging, independent of pharmacological modulation.

These findings have important clinical implications. First, the early divergence of SBP from DBP and rapid pulse pressure widening in individuals with obesity highlights the need for early, aggressive cardiovascular risk screening and intervention in this population [17, 31, 32]. Even in younger adults with obesity, SBP elevation and a widening pulse pressure may be indicative of subclinical vascular damage. Second, visualization of these age- and BMI-stratified trajectories can help enhance public and professional awareness of individualized vascular aging, illustrating that chronological age alone may underestimate the risk in those with obesity. Third, sex-stratified analyses underscore the importance of sex-specific hypertension management, especially in postmenopausal women who may experience abrupt changes in vascular stiffness [33, 34]. This also supports the integration of body composition and hormonal status into cardiovascular risk prediction models. The observed sex differences in the impact of obesity on blood pressure may be partly explained by hormonal and physiological mechanisms. Estrogen confers vascular protection in premenopausal women, delaying arterial stiffening compared with men, whereas its decline after menopause accelerates vascular aging, especially in those with excess adiposity. These biological differences highlight the need for tailored blood pressure management strategies that consider both sex and BMI. Furthermore, because obese individuals exhibited an earlier diastolic peak, indicating accelerated vascular aging, more frequent blood pressure screening and earlier lifestyle interventions may be warranted in younger adults with obesity to prevent or delay hypertension onset. In clinical terms, the earlier diastolic peak observed in obese individuals suggests accelerated vascular aging and highlights the need for earlier and more frequent blood pressure assessments in this population. Likewise, the earlier onset of pulse pressure widening in women—particularly those who are overweight—indicates that cardiovascular screening and preventive interventions should begin at a younger age in women compared with men [19]. Together, these findings support the development of individualized, sex- and BMI-specific monitoring strategies to optimize early detection and prevention of hypertension and vascular dysfunction.

Some of the strengths of this study include its large sample size, its collection of real-world data from standardized health checkups, and the use of flexible, non-parametric modeling to capture complex, nonlinear trajectories. The stratified analysis facilitated the exploration of detailed physiological interactions between age, sex, and BMI. However, this study also has several limitations that must be acknowledged, including the possibility that the findings may not be generalizable beyond Japanese populations, as the dataset was derived from a single health checkup facility and consisted of individuals who voluntarily participated in routine health examinations. Because some participants contributed multiple records across different years, repeated measurements from the same individual were treated as independent data points in the LOWESS analysis. This approach enabled visualization of continuous age-related trends at the population level; however, within-individual correlations were not explicitly accounted for. As a result, the trajectories should be interpreted as population-level smoothed patterns rather than subject-level longitudinal changes. In addition, the supplementary analysis excluding participants receiving antihypertensive medication was intended to confirm the robustness of the observed trajectories rather than to provide formal statistical comparisons. While the overall age-related patterns were largely consistent with those in the full dataset, potential selection bias should be considered, as untreated individuals may represent a healthier subgroup with different cardiovascular risk profiles. Furthermore, because the LOWESS method is a descriptive, non-parametric technique, it does not allow adjustment for potential confounders or provide confidence intervals that reflect uncertainty across different ages. These limitations are particularly relevant in older age groups, where data were sparser, and may also explain some residual confounding related to unmeasured factors such as smoking, diet, physical activity, socioeconomic status, or menopausal state. Implementing restricted cubic spline or other multivariable regression models in this dataset would require mixed-effects or generalized estimating equation frameworks to account for within-person correlation [35]. However, the present dataset was not originally designed as a longitudinal cohort, and repeated measurements were available for only a subset of participants at irregular intervals, making the application of spline-based mixed models technically infeasible and potentially biased. Moreover, the primary objective of this study was descriptive—to visualize population-level, sex- and BMI-stratified trajectories across the adult lifespan—rather than to estimate adjusted associations or test specific hypotheses. In this context, the use of LOWESS was considered most appropriate for capturing the overall shape of nonlinear hemodynamic trends without imposing model assumptions that may not hold in such a heterogeneous dataset. Future studies with standardized longitudinal follow-up and richer covariate information will be required to perform spline-based analyses to quantify these relationships more rigorously. In addition, because heart rate was derived from a single resting ECG, the potential influence of transient arrhythmias such as atrial fibrillation could not be fully excluded. Nevertheless, given the large sample size and population-based design, such effects are expected to be minor and unlikely to alter the observed age- and BMI-related trends. Furthermore, blood pressure was determined based on a single measurement at the time of the checkup rather than by home or ambulatory blood pressure monitoring. The analysis was also cross-sectional in design, thereby limiting the ability to make causal inferences. Furthermore, although the dataset covered a 13-year period, the trajectories presented in this study represent cross-sectional age-related patterns rather than longitudinal changes within the same individuals. Therefore, the curves should be interpreted as population-level differences across age groups rather than true temporal progressions. In addition, unmeasured lifestyle factors such as diet, stress, and medication adherence may have influenced the associations between BMI and blood pressure, and these factors were not available in the current dataset. Potential confounding factors such as smoking and physical activity were not adjusted for, and menopausal status data were unavailable. Additionally, the reliance on single-day measurements limits insights into both day-to-day variability and masked hypertension. Future longitudinal studies involving a repeated measures design and the use of vascular biomarkers (e.g., pulse wave velocity) are needed to validate and extend these findings. Interventional studies exploring the effects of lifestyle or pharmacological strategies on age-related hemodynamic changes may also identify critical windows for preventive care. Moreover, studies incorporating genetic and epigenetic data may shed light on individual susceptibility to accelerated vascular aging.

In conclusion, the results of this study highlighted the strong interactive effects of age, sex, and adiposity on vascular aging. The progressive increase in SBP, the DBP peak observed in midlife and the decline thereafter, and pulse pressure widening were more pronounced in individuals with obesity than in those without it and diverged according to sex. These findings emphasize the importance of BMI- and sex-specific approaches to cardiovascular risk stratification and the implementation of intervention strategies, and they also demonstrate the utility of trajectory-based visualization for public health communication and clinical guidance. Although the present study did not aim to define specific clinical thresholds or predictive models, the visualization of population-level trajectories can help identify high-risk subgroups and critical life stages for early intervention—particularly obese individuals and women around midlife, where hemodynamic changes appear to accelerate.

### Perspective of Asia

Population-wide health checkup systems are widely implemented in Japan and other Asian countries, providing repeated, standardized measurements of blood pressure and anthropometrics across a broad age range. This context makes descriptive, strata-specific visualization of hemodynamic aging particularly actionable for clinical practice and public health. In our Japanese dataset, SBP increased steadily with age, whereas DBP showed a midlife peak followed by a decline, and these patterns differed by sex and BMI category. Such sex- and adiposity-stratified trajectories may help clinicians interpret “age-appropriate” blood pressure components more precisely and identify subgroups in whom vascular aging appears accelerated (e.g., earlier DBP peak and earlier pulse pressure widening in higher BMI categories).

From an Asian guideline perspective, hypertension is commonly defined using thresholds consistent with the Japanese Society of Hypertension criteria, and obesity distributions and cardiometabolic risk profiles in East Asian populations differ from those in Western cohorts. Therefore, risk communication and screening strategies that incorporate both sex and BMI may be particularly valuable in Asia, where modest BMI elevations can still confer substantial cardiometabolic risk. Our findings support the potential utility of adopting sex- and BMI-tailored monitoring approaches in health checkup settings, including earlier preventive interventions for individuals with obesity and heightened attention to midlife transitions in women.
